# Optimal tolerability and high efficacy of a modified schedule of lapatinib–capecitabine in advanced breast cancer patients

**DOI:** 10.1007/s00432-013-1556-4

**Published:** 2013-11-29

**Authors:** T. Gamucci, L. Moscetti, L. Mentuccia, L. Pizzuti, M. Mauri, G. Zampa, I. Pavese, I. Sperduti, A. Vaccaro, P. Vici

**Affiliations:** 1 Medical Oncology Unit, ASL Frosinone, Via Armando Fabi, 03100 Frosinone, Italy; 2 Medical Oncology Unit, Belcolle Hospital, Strada Sammartinese 1, 01100 Viterbo, Italy; 3 Department of Medical Oncology B, Regina Elena National Cancer Institute, Via Elio Chianesi 53, 00144 Rome, Italy; 4 Oncology Unit, Department of Oncology, S. Giovanni-Addolorata Hospital, Via dell’Amba Aradam 9, 00184 Rome, Italy; 5 Oncology Unit, Nuovo Regina Margherita Hospital, Via Emilio Morosini 30, 00153 Rome, Italy; 6 Medical Oncology, San Pietro Hospital, Via Cassia 600, 00189 Rome, Italy; 7 Department of Biostatistics, Regina Elena National Cancer Institute, Via Elio Chianesi 53, 00144 Rome, Italy

**Keywords:** HER2-positive, Advanced breast cancer, Lapatinib, Capecitabine, Diarrhea, Schedule modification

## Abstract

**Purpose:**

Diarrhea in relation to the lapatinib–capecitabine regimen is a common and debilitating side effect which may interfere with optimal treatment delivery. We performed a post hoc analysis in human epidermal growth factor receptor 2-positive advanced breast cancer patients treated with a modified schedule in its administration, aimed primarily to evaluate grade (G) ≥2 diarrhea incidence and, secondarily, treatment efficacy.

**Patients and methods:**

Treatment schedule consisted of lapatinib 1,250 mg daily for the first 10 days, then in combination with capecitabine, 2,000 mg/m^2^, starting day 11 for the first cycle, and thereafter from day 8, for 14 days of a 21-day cycle, in 3 daily administrations. Lapatinib was dissolved in water, and cholestyramine was continuously given twice a day.

**Results:**

Among 38 patients treated and analyzed, the incidence of G ≥ 2 diarrhea was 13.2 %. In 28 patients diarrhea was not observed, while G1–2 diarrhea was reported in 9 (23.7 %) patients; a single episode of G3 diarrhea was observed in 1 (2.6 %) patient. Overall response rate was 34.2 %, clinical benefit 55.3 %, and median progression-free survival 10 months.

**Conclusion:**

The results of the present post hoc analysis are very encouraging, both in terms of tolerability and treatment efficacy, and all data compare favorably with previous reports of “conventional” administration of the lapatinib–capecitabine regimen.

## Introduction

Amplification of human epidermal growth factor receptor 2 (ErbB2 or HER2) occurs in approximately 20 % of breast cancers and is associated with poor prognosis. Trastuzumab, a monoclonal antibody toward the extracellular domain of HER2 receptor, combined with chemotherapy, increases time to progression and overall survival in advanced breast cancer patients. However, resistance to trastuzumab unfortunately is present or develops (Ross et al. 2009; Dawood et al. 2010).

Lapatinib, an orally available small molecule reversible tyrosine kinase inhibitor and an in vitro and in vivo potent selective dual inhibitor of ErbB1 (EGFR) and HER2 receptor, has been approved since 2007 in combination with capecitabine for treatment of metastatic breast cancer overexpressing HER2 and previously treated with anthracycline, taxane and trastuzumab. Lapatinib inhibits ErbB1 and HER2 intracellular kinase domains; it is known to be active in ErbB1 mutants and truncated forms of HER2 receptor (p95), and can overcome the resistance to trastuzumab (Blackwell et al. 2009; Burstein et al. 2008; Gomez et al. 2008; Iwata et al. 2006).

The association of lapatinib and capecitabine was evaluated in a phase III randomized trial (Geyer et al. 2006; Cameron et al. 2010), showing the efficacy of the combination treatment after trastuzumab failure, and the superiority over capecitabine alone in anthracycline and taxane pretreated advanced breast cancer patients. Among the most common adverse events observed in the registrative trial was diarrhea, representing the main limiting toxicity, occurring in more than a half of the patients in the combination arm (60 %), and partially reducing treatment compliance. Moreover, it was the most serious G3–4 adverse event [together with hand-foot syndrome, (HFS)], occurring in 12 % (G3) and 1 % (G4) of the patients, respectively (Geyer et al. 2006). In the expanded access program (LEAP), diarrhea was the most frequently reported drug-related serious adverse event (9.7 %) (Capri et al. 2010). Other clinical studies reported similar results, confirming diarrhea as the most common side effect occurring with lapatinib plus capecitabine regimen, requiring drug dose modification or treatment interruption in some cases (Crown et al. 2008). The frequency and severity of diarrhea, along with the not unusual long duration of the toxic effect, may limit full dosing and optimal treatment duration, possibly having an impact on treatment efficacy.

In clinical practice, outside of clinical studies, diarrhea in relation to lapatinib plus capecitabine treatment is a well-known side effect and, even if often of low grade, it is a common knowledge how it may lead to a reduced treatment compliance and a lower quality of life in treated patients.

Treatment guidelines for the management of lapatinib-associated toxicities (primarily diarrhea) are available (Crown et al. 2008; Benson et al. 2004; Moy and Goss 2007), and clinicians are now more capable of managing this toxic adverse event effectively in clinical practice, but diarrhea still represents an important limitation to the optimal regimen delivery in many patients.

In order to reduce the incidence and severity of the frequent gastrointestinal toxicity observed in patients treated with conventional schedule of lapatinib–capecitabine regimen, and to increase treatment compliance, a modified administration schedule was adopted. This consisted in administering capecitabine from day 11 instead of day 1 for the first cycle, then in subsequent cycles from day 8, and permanently dividing the planned capecitabine dose in three daily doses as a chronomodulated schedule as suggested by Santini et al. (2006). Moreover, lapatinib was dissolved in water, and cholestyramine was administered twice a day. This treatment schedule modification was applied to patients candidating for conventional lapatinib–capecitabine regimen. We recruited patients from nine Italian cancer centers, all treated with the above described modified administration schedule.

## Patients and methods

Our analysis comprises of HER2-positive advanced or metastatic breast cancer patient candidates for treatment with lapatinib–capecitabine. The HER2 status was considered positive if the local institution reported grade 3+ staining intensity (on a scale of 0–3) by immunohistochemical analysis, or grade 2+ staining intensity with gene amplification on fluorescence or chromogenic in situ hybridization. Patients recruited had an Eastern Cooperative Oncology Group (ECOG) performance status of 2 or less, a life expectancy of at least 12 weeks, a left ventricular ejection fraction (LVEF) within the institution’s normal ranges, and adequate organs and hematological functions.

The treatment schedule consisted of lapatinib, at a dose of 1,250 mg daily, 1 h before or after breakfast, administered as single agent for the first 10 days, then continuously, in combination with capecitabine, which was given at a dose of 2,000 mg/m^2^, starting on day 11 (for the first cycle), and then from day 8, for 14 days out of a 21-day cycle, and with a chronomodulated schedule (25 % of the dose at 8.00 a.m., 25 % at 12.00 a.m., and 50 % at 22.00 p.m.). According to the third amendment of ALLTO trial, lapatinib was always dissolved in water; furthermore, cholestyramine was administered, twice a day on a continuous basis, long after capecitabine and lapatinib intake.

Standard efficacy and toxicity evaluations were performed in all the patients treated, paying special attention to diarrhea incidence and severity.

The number and duration of diarrhea episodes were reported by patients in a personal diary. Treatment was given until disease progression or unacceptable toxic effects. Standard recommendations for capecitabine and lapatinib dosage modifications were followed for the management of adverse events. The primary endpoint of the analysis was the evaluation of the tolerability of treatment in terms of diarrhea G ≥ 2 incidence and of the patients’ compliance; secondary end points were treatment efficacy evaluation, in terms of overall response rate (ORR), clinical benefit (CB, responses and stable disease for at least 6 months), response duration, progression-free survival (PFS), and overall survival (OS).

### Statistical analysis

Descriptive statistics were used to summarize pertinent study information. The association between variables was tested by the Pearson’s Chi-square test or Fisher’s exact test, when appropriate. Survival curves were calculated by the Kaplan–Meier product-limit method from the treatment starting date until the time of death (OS), progression (PFS), or last visit (OS and PFS), whichever applicable. SPSS software (SPSS version 20.0, SPSS Inc., Chicago, Illinois, USA) was used for all statistical evaluations.

## Results

From November 2010 to December 2012, 38 HER2-overexpressing advanced or metastatic breast cancer patients were treated with the modified schedule of lapatinib–capecitabine regimen in 9 Italian cancer centers. Main patient and tumor characteristics are reported in Table 1. Twenty-one patients received adjuvant chemotherapy, in 13 patients combined or followed by adjuvant trastuzumab; the remaining 8 patients received chemotherapy without trastuzumab as adjuvant treatment. Ten patients received neoadjuvant treatment, in 9 patients including trastuzumab (one patient chemotherapy only). Eight patients received adjuvant endocrine treatment for 5 years, concomitantly to trastuzumab for 6 months; 15 patients received endocrine treatment in combination with trastuzumab for advanced disease. In regard to chemotherapy, the median number of previous chemotherapy lines was 1 (76.3 %), ranging from 1 to 4 overall. All of the patients had previously been treated with trastuzumab, ranging from 1 to 4 previous lines, including neoadjuvant and adjuvant setting, but most of the patients (78.9 %) had received only one prior chemotherapy regimen containing trastuzumab for advanced disease. The median number of cycles of lapatinib–capecitabine modified schedule delivered was 7 (range 2–21). Altogether, 330 cycles were administered. Ten women (26.3 %) received more than 10 cycles of therapy.

**Table 1:** Patient characteristics (*N* = 38)

Characteristics	*N* (%)
Median age, years (range)	55 (41–84)
ECOG PS, median (range)	1 (0–1)
ER/PR status, *n* (%)	
ER+ and/or PR+	15 (39 %)
Prior chemotherapy regimens, median (range)	1 (1–4)
Prior trastuzumab therapy, *n* (%)	
Neoadjuvant	9 (23.6 %)
Adjuvant	13 (34.2 %)
Metastatic	38 (100 %)
Number of prior trastuzumab lines, median (range)	1 (1–4)
Prior adjuvant hormonal therapy, *n* (%)	8 (21 %)
Prior hormonal therapy for advanced disease, *n* (%)	15 (39 %)
Cycles of lapatinib and capecitabine delivered, median (range)	7 (2–21)

Overall, diarrhea events were low grade, neither requiring lapatinib nor capecitabine dose modification nor interruption. None of the patients discontinued or reduced lapatinib dose for any reason when lapatinib was given as single agent in the first 10 days of treatment. Overall, in 312/330 (94.5 %) cycles administered and in 28/38 patients, no treatment-related diarrhea was observed; 5 patients (13.2 %) experienced G1 diarrhea (11/330 cycles, 3.3 %), and 4 patients (10.5 %) experienced G2 diarrhea (6/330 cycles, 1.8 %), while G3 diarrhea was observed in only 1 (2.6 %) patient in the second cycle (1/330 cycles, 0.3 %). No episodes of G4 diarrhea were recorded (Table 2). Overall, the incidence of G2 or more severe diarrhea in our analysis was 13.2 %. No capecitabine or lapatinib diarrhea-related discontinuation was observed, and in the only one patient experiencing a single episode of G3 diarrhea, a 25 % capecitabine dose-reduction was performed for 2 weeks, rapidly improving the symptom. In regard to other toxicities, cutaneous toxicity was the only side effect leading to dose reduction and/or treatment discontinuation (Table 3). Three patients had to reduce capecitabine dose use due to HFS, after 7, 9, and 10 cycles, respectively, and in 1 patient, capecitabine was definitively discontinued due to G3 HFS, while lapatinib was continued as monotherapy until disease progression. Other relevant toxicities observed were rash and ungueal alterations, with no cases of rash G3/G4, 2 (5.3 %) patients experiencing transient G1 rash, and 7 (18.4 %) patients G2 rash, after more than 8 cycles, with only 1 patient discontinuing capecitabine for 2 weeks, with a prompt resolution of the symptom. Ungueal alterations, usually mild, occurred in 6 patients, and were G1 in 1 patient and G2 in 5 patients. Other toxicities were in the range of reported literature data. We did not observe any clinical cardiotoxicity, nor significant decreases of LVEF. No toxic deaths were recorded.

**Table 2:** Diarrhea incidence and severity

Grade	*N* (%)
1	5 (13.2 %)
2	4 (10.5 %)
3	1 (2.6 %)
4	–

**Table 3:** Cutaneous toxicity in 38 patients (%)

Toxicities	G1	G2	G3	G4
Rash	2 (5.3 %)	7 (18.4 %)	–	–
Hand-foot syndrome (HFS)	1 (2.6 %)	3 (7.9 %)	1 (2.6 %)	–
Ungueal alterations	1 (2.6 %)	5 (13.1 %)	–	–

**Fig. 1:**
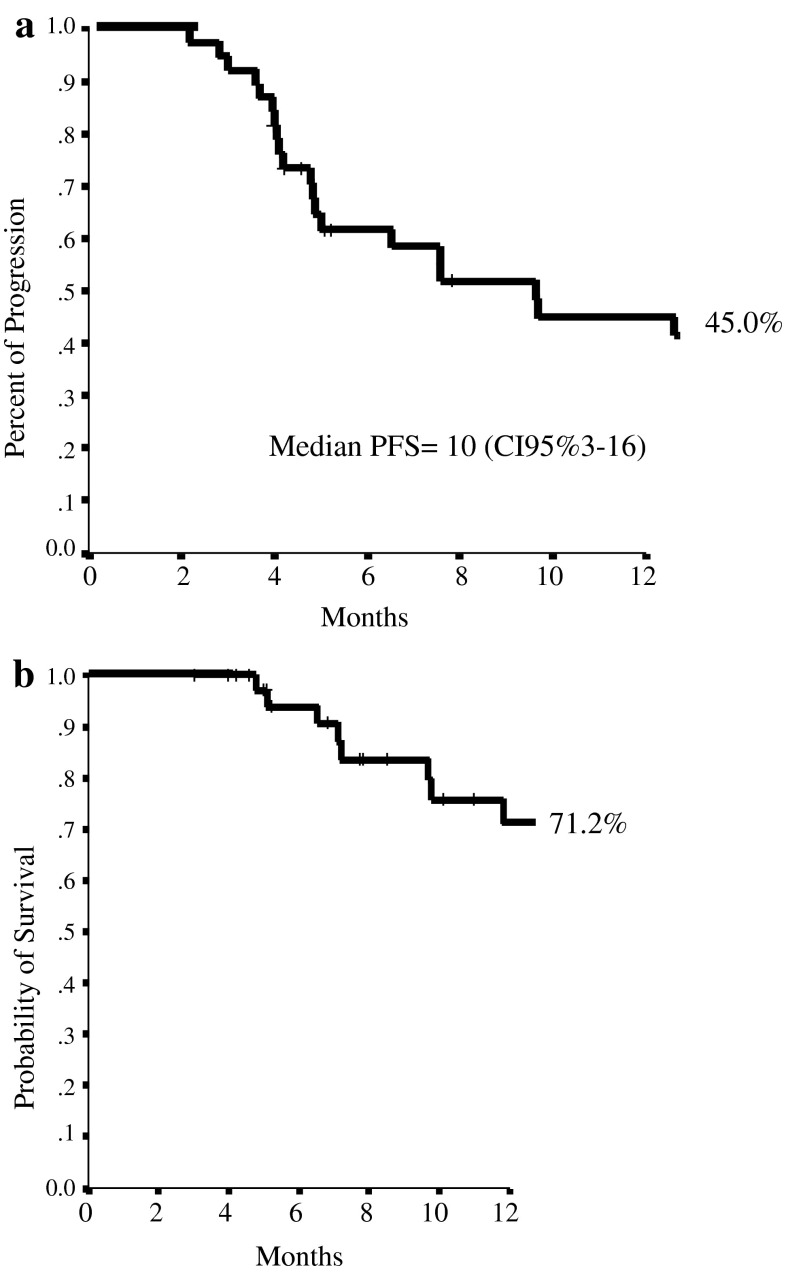
**a** 1-year progression-free survival (PFS). **b** 1-year overall survival (OS)

As treatment efficacy concern (Table 4), 2 patients (5.3 %) achieved a complete response (CR) and 11 patients (28.9 %) a partial response (PR), for an ORR of 34.2 % (95 % CI 19.1–49.3); 20 patients (52.6 %) had stable disease (SD) and 5 patients (13.2 %) progressive disease (PD). CB was observed in 21 patients (55.3 %). The median duration of response was 16 months (95 % CI 4–21). The median PFS was 10 months (95 % CI 3–16), and 1-year PFS was 45.0 % (Fig. 1a). After a median follow-up of 10 months, (range 3–20), 1-year OS was 71.2 %, with 17 (44.7 %) patients surviving more than 12 months (Fig. 1b). At the time of data collection, seven patients were still on treatment.

**Table 4:** Summary of clinical efficacy

	Patients (*N* = 38)
ORR	
CR or PR confirmed, % (95 % CI)	34.2 % (19.1–49.3)
CBR	
CR or PR or SD ≥ 24 weeks, % (95 % CI)	55.3 %
Overall PFS	
Progressions *n* (%)	31 (81.5)
Censored^a^ * n* (%)	7 (18.5)
Median PFS, months, % (95 % CI)	10 (3–16)
1-year PFS, %	45
1-year overall survival, %	71.2

*ORR* overall response rate, *CR* complete response, *PR* partial response, *CBR* clinical benefit rate, *PFS* progression-free survival

^a^ Patients who did not die or progress until the clinical cutoff for these data (May 31, 2013)

## Discussion

Despite the existence of treatment guidelines for the management of lapatinib–capecitabine-associated diarrhea, it still represents a significant limitation in the optimal regimen administration in many patients. This frequently has a negative impact on patients’ quality of life, where the dose reduction, interruption, and discontinuation of treatment may negatively influence the efficacy in daily clinical practice.

In the present analysis, we intended to assess whether a change in the timing and mode of administration of lapatinib–capecitabine regimen, in combination with support medication, could lead to a reduction in treatment-related main toxicities, with a particular focus on diarrhea. Our results satisfy a post hoc statistical hypothesis according to A’Hern exact single-stage phase II design (2001), considering a percentage of diarrhea of grade ≥2 higher than 35 % (10) unacceptable, and assuming levels ≤15 %, with a significance of 5 % and a power of 90 %, interesting.

Although lapatinib provides a new treatment option for the management of HER2 positive breast cancer patients, and the combination with capecitabine represents an active and consolidated treatment choice in trastuzumab-resistant disease, clinicians and patients still face a number of clinical challenges, including minimizing toxicity. In fact, toxic effects of cancer treatments are one of the main limitations among planned dose-intensity maintenance, as well as having a relevant impact on costs, and on quality of life, which is of paramount importance in the advanced stages of the disease, where a satisfactory quality of life represents one of the major goals of the treatments. Moreover, patient non-adherence to oral antineoplastic therapy, especially if not well tolerated, is a well-known barrier to treatment effect. The search for alternative drug administration schedules that allow to reduce the toxic effects of standard therapy is a major challenge to make the patients to be able to assume adequately therapy, and to improve their quality of life.

Gastrointestinal side effects, mainly diarrhea of any grade, are described in the literature data in more than a half of the patients with lapatinib as single agent (Crown et al. 2008). The data from the trial of Gomez show, for lapatinib 1,500 mg daily as monotherapy, an incidence of diarrhea of 46 %, usually mild, being of grade 3 only in 1 % of patients (2008). In the ALLTO adjuvant trial, diarrhea of any grade was reported in 61 % of patients, being of grade 3–4 in 6 % of patients in the lapatinib arm (Goss et al. 2013). In regard to the use of lapatinib in combination with antineoplastic agents other than capecitabine, phase III trials in neoadjuvant setting showed a higher incidence of treatment interruptions in the arms containing lapatinib, mainly due to diarrhea. The NeoALTTO trial reports 21 % of grade 3 diarrhea in the lapatinib arm, whereas in the CherLob trial, grade 3–4 diarrhea was recorded in 37 % of patients (in association with chemotherapy) (Baselga et al. 2012; Guarneri et al. 2012). In the GeparQuinto neoadjuvant trial, diarrhea of any grade was observed in 75 % of the patients, being of grade 3–4 in 11.7 % of the patients (Untch et al. 2012).

The combination of lapatinib and capecitabine in the phase III registrative trial determined grade 3 diarrhea in 12 % of patients, with 1 % of patients experiencing grade 4 diarrhea, while mild (grade 1–2) diarrhea occurred in 47 % of patients. These effects led to treatment discontinuation in 3 % of patients, while delays or dose reductions have been reported in 11 % and 5 % of patients, respectively (Geyer et al. 2006; Crown et al. 2008). In a pooled analysis of nine phase I–II–III trials evaluating diarrhea, in which lapatinib was administered at doses ranging from 1,000 to 1,500 mg daily as a single agent or in combination with capecitabine or taxanes, diarrhea occurred in 54 % of lapatinib-treated patients and in 24 % of patients not receiving lapatinib. In more detail, diarrhea of any grade was reported in 51 % of the patients treated with lapatinib as a single agent, in 65 % of patients treated with lapatinib plus capecitabine, and in 48 % of patients treated with lapatinib plus a taxane. The symptom was usually mild to moderate, being of grade 3 in less than 10 % of patients, and of grade 4 in less than 1 % of patients. Moreover, grade 3 diarrhea in 10–17 % of the patients treated with 1,500 mg once daily was reported, whereas this percentage increased to 21 % in patients treated with 750 mg BID, suggesting an association with dose and schedule. In regard to timing of symptom development, approximately 40 % of patients developed diarrhea within the first 6 days of treatment. In addition, each diarrhea episode lasted a mean of 7–9 days, and most (81 % in the lapatinib–capecitabine group) diarrhea events were resolved without dose-modification and with conventional approaches, even if 16 % of patients had to reduce doses or delay treatment, with 3 % discontinuing treatment (Crown et al. 2008).

In order to improve gastrointestinal tolerability of lapatinib–capecitabine regimen in advanced breast cancer, other sporadic experiences with treatment schedule modification were carried out. A recently published report on lapatinib–capecitabine schedule modification, consisting of a 7 day capecitabine intake followed by a 7 day rest, reported satisfactory activity and an improvement in gastrointestinal toxicity, with no cases experiencing G3–4 diarrhea, with G2 diarrhea occurring in 26 % of patients (Gajria et al. 2012).

In our experience, the difficulty in successfully managing of diarrhea often limiting patients compliance in terms of treatment adherence and a decreased quality of life has been addressed by delaying the administration of capecitabine in the first cycle of treatment, which was introduced in a chronomodulated manner when lapatinib was administered as a single agent after a 10-day time frame. At the same time, lapatinib was dissolved in water, and cholestyramine was given after lapatinib consumption to counter possible drug interference. These schedule modifications were successfully tolerated by most patients. In fact, we observed a very high adherence to therapy administered, with a median of 7 cycles delivered and 10 patients (26.3 %) receiving more than 10 cycles, observing a particularly low incidence of grade 2 diarrhea (10.5 %), with only one episode of grade 3, and no episodes of grade 4 diarrhea. The incidence of diarrhea of grade ≥2 in the present experience (13.2 %) compares favorably with conventional lapatinib plus capecitabine regimens in the literature data, reporting an incidence of approximately 33 % (Geyer et al. 2006; Crown et al. 2008). Moreover, the efficacy of lapatinib–capecitabine treatment observed in our patient population was very encouraging, with an ORR of 34.2 %, a CB of 55.3 %, a median duration of response of 16 months, and a median PFS of 10 months. These compare favorably with other results of lapatinib–capecitabine regimens administered with standard schedules, reporting response rates ranging from 22 to 30.8 %, a median response duration of about 6.5 months, and a median PFS ranging from 5.0 to 8.4 months (Geyer et al. 2006; Capri et al. 2010; Verma et al. 2012). Moreover, in our experience, treatment was safely tolerated for a long period in many patients, confirming its optimal tolerability. Whether this consistent improvement in tolerability and the related toxicities increased patients compliance may have favorably influenced regimen efficacy is difficult to demonstrate, but the characteristics of patients in the present analysis do not differ from those enrolled in other published trials, and the possible effect on treatment efficacy cannot be excluded either.

On the whole, even taking into consideration the limitations of a post hoc analysis of a subgroup of patients treated across different cancer centers, our experience with the above reported modified schedule of lapatinib–capecitabine regimen in HER2 positive advanced breast cancer patients can be considered a very satisfactory experience, with encouraging treatment efficacy results and, above all, an optimal quality of life in the treated patients.

## Conclusions

Our experience of applying the modified schedule of lapatinib–capecitabine regimen led to a significant reduction in the incidence and severity of treatment-related diarrhea, allowing optimal delivery of chemotherapy with encouraging efficacy data, while maintaining a good quality of life in the majority of patients treated.
